# Promoting Intergenerational Health in Rural Kentuckians With Diabetes (PIHRK’D): Protocol for a Longitudinal Cohort Study

**DOI:** 10.2196/69301

**Published:** 2025-07-24

**Authors:** Brittany L Smalls, Courtney L Ortz, Makenzie Barr-Porter, Heather Norman-Burgdolf, Christopher J McLouth, Brittany Harlow, Oluwatosin Leshi

**Affiliations:** 1 Department of Family Medicine College of Medicine University of Kentucky HealthCare Lexington, KY United States; 2 Department of Dietetics and Human Nutrition Martin-Gatton College of Agriculture, Food, and Environment University of Kentucky Lexington, KY United States; 3 Department of Biostatistics College of Public Health University of Kentucky Lexington, KY United States

**Keywords:** type 2 diabetes mellitus, type 2 diabetes, intervention, family-based, physical activity, nutrition, rural, social support, self-care

## Abstract

**Background:**

The prevalence of type 2 diabetes mellitus (T2DM) is steadily increasing and has exceeded 20% in some rural Kentucky counties. In Kentucky, chronic diet-sensitive conditions and unhealthy behaviors are among the highest in the nation, with approximately 36.5% of adults with obesity and 13.3% diagnosed with T2DM, while only 15.3% meet physical activity recommendations, and 4.7% meet fruit and vegetable consumption recommendations. Family-based interventions can be used to promote health in rural communities that often comprise intergenerational households.

**Objective:**

The purpose of the study is to determine whether leveraging family units as sources of social support promotes nutritional and physical activity changes among those who are overweight or have obesity and are diagnosed with T2DM.

**Methods:**

This study consists of 3 phases (baseline, intervention, and postintervention). An overview of the study will be provided to interested participants, after which their consent will be sought. At baseline, demographic data, social support, physical activity, diabetes knowledge, diabetes self-management, dietary recall, obstructive sleep apnea, and sleep health, as well as social network data, will be collected using validated questionnaires. Anthropometric (weight, height, lean mass, and body fat) and T2DM-related (blood pressure, glycated hemoglobin A_1c_ [HbA_1c_], total cholesterol, low-density lipoproteins, high-density lipoproteins, and triglycerides) clinical measures will also be obtained. During the intervention phase, participants will complete 6 months of medical nutrition therapy (MNT) alongside 1 member of their social network (eg, household member). In addition, participants will have the option to attend Dining with Diabetes program sessions that are offered at the county level through Cooperative Extension Service (Extension) agents. Follow-up data collection will include clinical measures, a dietary recall, and the assessment of participants’ stage of behavior change at 3, 6, 9, and 12 months postintervention.

**Results:**

This study was funded in November 2022 by the American Diabetes Association (ADA). Data collection started on September 1, 2023, and is projected to end in November 2025. We have enrolled a total of 48 participants in the study.

**Conclusions:**

T2DM is a growing problem, particularly among vulnerable populations in rural Kentucky. This study plans to leverage family units as sources of social support with MNT and nutrition education through the Dining with Diabetes program. If effective, this approach will inform future family-based interventions in rural communities.

**Trial Registration:**

ClinicalTrials.gov NCT06080425; https://clinicaltrials.gov/study/NCT06080425

**International Registered Report Identifier (IRRID):**

DERR1-10.2196/69301

## Introduction

### Background

Type 2 diabetes mellitus (T2DM) and obesity have a compounding effect on health disparities in rural communities in Kentucky. T2DM is a persistent public health condition affecting 34.1 million US adults [1]. Moreover, geographic disparities exist with some population segments, such as rural-dwelling Americans who experience greater vulnerability to this condition. The prevalence of T2DM in Kentucky’s rural counties has reached 23%, compared to the overall state prevalence of 13.8% [2]. Moreover, in Kentucky, obesity and related conditions are among the highest in the nation, with approximately 40% of adults classified as obese, 40% with hypertension, and 38% with high cholesterol, while 30% meet physical activity recommendations, and 8.9% meet fruit and vegetable consumption recommendations [2,3].

The prevalence of T2DM increases with age, and an estimated 25% of older adults (≥65 years old) have T2DM [3,4]. The increased prevalence of T2DM among older adults is consistent with the increased prevalence of T2DM where an estimated two-fifths of older adults have obesity [5]. Even though residents in rural Kentucky communities are disproportionally affected by obesity and T2DM, they have poor social determinants of health, which contributes to and sustains poor health outcomes.

Environmental and behavioral factors are possible contributors to intergenerational obesity and T2DM in rural Kentucky communities. The literature indicates that patterns of obesity can be seen within families [6,7]. These patterns are thought to be due to the environments where people live, work, and play [8]. These environmental factors influence individuals’ relationships with food within the family unit. Individuals raised in homes where unhealthy relationships with food have been developed are more likely to repeat these behaviors, thus increasing the potential for weight gain and obesity throughout the lifespan. Many of those with T2DM have at least 1 family member also with the condition, exhibiting a pattern of inheritance [9,10]. In addition, the likelihood of developing T2DM is 40% if one parent has T2DM and 70% if both parents have been diagnosed [11]. Familial links to diabetes via certain genetic factors and epigenetic changes have been noted [11]. However, those changes are mostly attributed to the environment and individuals’ behaviors, demonstrating the complex relationship between gene expression and the environment. Specifically, T2DM has been linked to risk factors, such as unhealthy eating, a sedentary lifestyle, and stress [11]. Though gene expression modifications occur via epigenetics, those changes can be reversible through environment and behavior modifications [11].

Family-based interventions can be used to promote healthy and beneficial lifestyle behaviors [12]. With an estimated 59.7 million US residents living in multigenerational households [13], it is imperative we understand how this family structure impacts the health of its members. This type of family unit is particularly of interest in rural Appalachia since families share cultural health beliefs and health behaviors. In addition to intergenerational households, many families in rural eastern Kentucky live on the same piece of land passed through generations. Family-centered behaviors [14,15] have consistently influenced nutrition and overall health outcomes positively [16]. As disease management predominately occurs within the home, use of family-based interventions related to adult chronic diet-sensitive diseases have been associated with positive health-related outcomes [17-19]. Secure, supportive relationships with family or close friends improve personal management of these conditions. Thus, developing an intervention that enhances social support and mitigates environmental factors that hinder nutrition and physical activity is imperative for sustained behavior change.

The burden of T2DM and obesity can be alleviated with common self-care activities, specifically healthy eating and physical activity. This combination of self-care practices has been shown to be more effective than either alone. Both nutritional changes and increased physical activity are factors contributing to weight loss, which is an important aspect of obesity and T2DM management that improves health outcomes and reduces long-term health complications [20]. However, residents of rural Appalachian communities report inadequate diet and physical activity behaviors, a lack of access to nutritious foods and opportunities for physical activity, geographical isolation from health-promoting resources, and poorer health outcomes than the rest of the nation [21].

This project is informed by the National Framework for Health Equity and Well Being [22], which was recently developed by the Cooperative Extension Service (Extension), a trusted community entity identified as a critical resource to address health inequities in rural communities with limited infrastructure to support public health [23]. This framework explicitly acknowledges the multiple levels of influence on health outcomes and the role of the Extension as a mitigator of community-level health disparities and draws attention to health inequities at various societal levels, including root causes of structural inequity; norms, policies, and practices; and social determinants of health. As individuals flow through multiple sectors of environments that they live in, each has a direct influence individually and collectively. This research specifically focuses on how county-level Extension agents (federally mandated program) can be used to mitigate health disparities that contribute to intergenerational obesity and T2DM management in rural Kentucky. Community-level factors also impact health outcomes, such as a lack of access to nutritious, affordable food, as well as availability of health-related resources. In this study, community assets will be gathered using subjective and objective community audits and assessed at the participant level using social network analysis. Societal-level factors include social norms and cultural health beliefs that impact health decision-making within the community, particularly at the interpersonal level of families and households.

Extension has a wealth of research- and practice-based programs that assist individuals throughout the lifespan to facilitate lifestyle changes that reduce chronic diet-sensitive conditions. As the largest providers of nutrition education in the country, Extension focuses on healthy eating, food security, physical activity, and obesity prevention [24]. This project intends to use the 4-session Dining with Diabetes education program offered in more than 25 states, including Kentucky. This program is nationally recognized, aligns with the Association of Diabetes Care and Education Specialists (ADCES) 7 Self-Care Behaviors, and increases self-efficacy for T2DM self-management [25-27]. This paper describes the protocol of Promoting Intergenerational Health in Rural Kentuckians With Diabetes (PIHRK’D), an intervention to determine whether leveraging family units as sources of social support promotes nutritional and physical activity changes among individuals with both overweight/obesity and T2DM and also to assess whether members of the household who engage in the intervention will show changes in anthropometric measures for an at-risk weight/body composition or maintain a healthy body composition.

### Study Aims

The following are the aims of this study:

- Aim 1: Use social network analysis to describe (1) community assets (eg, access to healthy eating and ways to participate in physical activity) and (2) intergenerational links to obesity and diabetes (eg, parent, sibling, child).
- Aim 2: Deliver a tailored, household-specific nutrition therapy and physical activity plan.
- Aim 3: Determine the preliminary efficacy of tailored nutrition therapy and physical activity for those living within the household. The hypotheses for the third aim are that (1) participants (individuals with overweight/obesity and diabetes) will have a clinically meaningful change in HbA_1c_ (≥0.5%) and weight (≥–6%) and (2) members of the households will show improved anthropometric indicators of overweight/obesity or maintain a healthy weight.

## Methods

### Study Design

This longitudinal cohort study has been registered at ClinicalTrials.gov (NCT06080425; October 3, 2023). The study will test the feasibility, acceptability, and preliminary effectiveness of the proposed intervention. Analysis will be conducted at the individual level for the enrolled participants with T2DM and at the household level via cluster analysis. Study activities will occur in participants’ homes and local Extension offices.

### Ethical Considerations

The study was approved by the Institutional Review Board (IRB) of the University of Kentucky (approval number 83972; approval date August 1, 2023). Research staff members will obtain written informed consent from all participants meeting the inclusion criteria prior to study enrollment.

The principal investigator (PI) will ensure that all research personnel undergo Collaborative Institutional Training Initiative (CITI) training for conducting human subject research and gaining access to study data. The PI will monitor the study to ensure that all potential risks to participants remain minimal. In addition, all research personnel will be provided with a standard operating procedure manual that outlines recruitment strategies; the informed consent process, including study procedures, contact information for the University of Kentucky Office of Research Integrity, and the PI, study timeline, and an appendix of required data collection documents; and a copy of the most recent consent forms and IRB study approval letter.

Measures will be put in place to protect the confidentiality of participants and the data to be collected in this study. All data, including health information, will be deidentified to ensure the anonymity of the study participants and will be stored on password-protected servers at the University of Kentucky. To track participation over time, an enrollment log that includes participants’ study IDs will be created, and only the study IDs (eg, deidentify) will be used when entering data into REDCap. Access to the study data will be restricted to authorized personnel directly involved in the research who will adhere to strict confidentiality protocols . Data and electronic records will be retained in accordance with regulatory and ethical standards for a minimum of 6 years post–study closure and then will be disposed of securely. All precautions will be taken to keep participants safe. Participants’ personal information will be safeguarded, and explicit consent will be obtained before disclosing any potentially identifying information. To guard against a potential breach of confidentiality, all study files will be deidentified and kept on a password-protected secure server at the University of Kentucky. Study-related findings will be presented at scientific meetings and published in peer-reviewed journals. The research will also commit to full compliance with data protection laws, including the Health Insurance Portability and Accountability Act (HIPAA), reflecting an unwavering dedication to upholding the highest ethical and legal standards, while preserving the privacy and security of all participants.

The PI will invite the Office of Research Integrity to conduct mock audits on data collection and deidentification, encryption of audio files, and informed consent documentation to ensure research participants are properly protected. We will inform participants if we learn new information that could change their mind about continuing with the study. We may ask participants to sign a new consent form if the information is provided after they have enrolled in the study.

For each data collection time point that the participants complete, they will receive US $30 (baseline, 3 and 6 months, and 3 and 6 months postintervention). In addition, those participants who attend at least 3 of 4 sessions of Dining with Diabetes with a family member will receive a US $50 gift card. Family members who consent to participate and accompany participants will not receive any payment.

### Eligibility Criteria

Community members interested in participating will be screened for eligibility to include adults (≥18 years old), diagnosed with T2DM, with more than 1 person in the household, and having their primary residence in the selected rural counties for at least 1 year. Adolescents (10-17 years old) will be permitted to participate in the study activities as family members but not as the main study participants. A trained research coordinator and research dietitian will obtain informed consent.

### Intervention

The intervention phase of this feasiblity study consists of a 6-month nutrition therapy and physical activity intervention. The main component of this study is MNT, which will be used to tailor healthy eating and physical activity. Specifically, MNT has been shown to be an effective strategy to manage T2DM [28]. A research dietitian will conduct monthly in-home MNT sessions for members of the household, but the MNT will be tailored for participants who have overweight/obesity and T2DM. The research dietitian will meet with the household or at least the enrolled participant with T2DM monthly for 6 months. Dietary counseling will use motivational interviewing approaches and will focus on living with T2DM, appropriate and feasible food choices, glycemic control, the importance of weight management, and goal setting. In addition, the household will be provided with American Diabetes Association (ADA)-endorsed physical activity recommendations tailored to the participants’ physical ability and their availability and access to opportunities for physical activity. The research dietitian’s notes will be maintained for each session during the intervention. Notes will be maintained in the standardized framework of the Nutrition Care Process and the modified assessment, diagnosis, intervention, monitoring, and evaluation (ADIME) format.

As a supplement to MNT counseling and activity recommendations, participants will enroll in the group-facilitated Dining with Diabetes program at their local Extension offices. Participants will be encouraged to have a member of their household attend the Dining with Diabetes sessions with them in an effort to promote and reinforce healthy lifestyle choices. This program will be offered free of charge at the county Extension offices. Dining with Diabetes is an interactive 4-session program that includes direct education on healthy eating, food preparation strategies, physical activity, emotional health, identifying complications of diabetes, and problem-solving skills in a group setting. Each session provides participants with an opportunity to learn how to prepare and taste diabetes-appropriate recipes. Like MNT, each session of Dining with Diabetes encourages participants to set and discuss diabetes management-related specific, measurable, achievable, relevant, and time-bound (SMART) goals to improve overall health outcomes and address challenges faced with diabetes management. Existing evaluation tools for the Dining with Diabetes program will provide secondary data for this study. The pre- and postevaluation tools for this program measure knowledge gained related to food choices and physical activity, as well as motivation and confidence in managing diabetes. A 3-month follow-up evaluation is also provided with the program that measures sustainable behavior change related to food choices, preparing meals at home, and physical activity patterns. This program will be administered by Extension agents bimonthly, for 8 weeks, starting in month 2 of participant enrollment.

Extension agents are trained to offer Dining with Diabetes, with many choosing to regularly offer this program in their communities. This program was developed to be implemented by Extension, and previous studies have documented the effectiveness of the program in improving overall health metrics (eg, glycated hemoglobin A_1c_ [HbA_1c_], hypertension) and increasing self-efficacy for diabetes self-management using Extension as a delivery mechanism [25,26]. See Table 1 for the schedule of enrollment, interventions, and assessments.

**Table 1:** Schedule of enrollment, interventions, and assessments.

Time points		Enrollment study period			Intervention study period								Postintervention study period		
		–t_1_	Baseline	t_1_		t_2a_	t_2b_	t_3a_	t_3b_	t_4_	t_5_	t_6_		t_9_	t_12_
**Enrollment**															
	Eligibility screen	X^a^	—^b^	—		—	—	—	—	—	—	—		—	—
	Informed consent	—	X	—		—	—	—	—	—	—	—		—	—
**Intervention**															
	MNT^c^	—	—	X		X	—	X	—	X	X	X		—	—
	Dining with Diabetes	—	—	—		X	X	X	X	—	—	—		—	—
**Assessments**															
	HbA_1c_^d^ and blood lipids	—	—	X		—	—	X	—	—	—	X		X	X
	Height	—	—	X		—	—	X	—	—	—	X		X	X
	Body weight	—	—	X		—	—	X	—	—	—	X		X	X
	Body fat	—	—	X		—	—	X	—	—	—	X		X	X
	Lean mass	—	—	X		—	—	X	—	—	—	X		X	X
	BMI	—	—	X		—	—	X	—	—	—	X		X	X
	Blood pressure	—	—	X		—	—	X	—	—	—	X		X	X
	24-hour dietary recall	—	—	X		X	—	X	—	X	X	X		X	X
	Social network data	—	X	—		—	—	—	—	—	—	—		—	—
	Demographics	—	X	—		—	—	—	—	—	—	—		—	—
	IPAQ^e^	—	X	X		—	—	X	—	—	—	X		X	X
	MOS^f^ Social Support Survey	—	X	X		—	—	X	—	—	—	X		X	X
	The Friends & Family Involvement in Adults’ Diabetes	—	X	X		—	—	X	—	—	—	X		X	X
	DDS^g^	—	X	X		—	—	X	—	—	—	X		X	X
	Diabetes Empowerment Scale	—	X	X		—	—	X	—	—	—	X		X	X
	DKQ^h^	—	X	X		—	—	X	—	—	—	X		X	X
	DSMQ^i^	—	X	X		—	—	X	—	—	—	X		X	X

^a^Applicable.

^b^Not applicable.

^c^MNT: medical nutrition therapy.

^d^HbA_1c_: glycated hemoglobin A_1c_.

^e^IPAQ: International Physical Activity Questionnaire.

^f^MOS: Medical Outcomes Study.

^g^DDS: Diabetes Distress Scale.

^h^DKQ: Diabetes Knowledge Questionnaire.

^i^DSMQ: Diabetes Self-Management Questionnaire.

### Outcomes

Anthropometry and point-of-care measures will be collected from participants at each data collection time point. Both the research dietitian and the research coordinator will be trained and will complete interrater reliability testing to complete data collection. Participants’ height will be assessed through a portable Seca stadiometer. Their body weight, body fat, lean mass, and BMI will be collected via the Tanita DC-240 Total Body Composition Analyzer. Their blood pressure will be measured with the ADC e-sphyg Digital Aneroid Sphygmomanometer. Cholestech LDX (Cholestech Corp., Hayward, CA) and HbA_1c_ Now+ Analyzers will be used to measure blood lipids and HbA_1c_, respectively.

### Sample Size

Power analysis for aim 3 was based on a total of 75 recruited participants, with an 80% retention rate, resulting in an analytic sample of 60 participants. The detectable effect size for a change in the primary outcome over time was Cohen f=0.14 at 80% power with 2-sided α=.05. This model assumes that a completely within-subject design with 5 testing occasions a first-order autocorrelation where r=0.5. Thus, with a total sample size of 60 participants, we would be powered to detect a small-to-medium effect.

### Recruitment

Recruitment started on September 1, 2023, and will be conducted via Extension offices, local media (eg, newspaper, radio public service announcement [PSAs]), word-of-mouth, social media, University of Kentucky Healthcare outpatient clinics (eg, internal medicine, family medicine, endocrinology), and the United Kingdom’s Barnstable Brown Diabetes and Obesity Center. Participants will be screened by the research dietitian to confirm obesity/overweight and T2DM diagnosis and identify each participant’s placement within the Transtheoretical Model (Stages of Change): precontemplation, contemplation, preparation, action, maintenance, or relapse [29]. The Enrollment Stage of Change will be used to develop appropriate goals for each participant. The research coordinator will enroll the participants in the study. Beyond the primary enrolled participants, members of the participants’ households will be invited to attend meetings with the dietitian and Dining with Diabetes program sessions. Household members will be screened for eligibility to include adults (≥18 years old), a diagnosis of T2DM, and the primary residence in the selected rural counties for at least 1 year. Adolescents aged 10- 17 years will be permitted to participate in the study activities as household members and not as the main study participants. To enhance participants’ buy-in and sustainability, the research dietitian will use motivational interviewing principles [30] to work with them to improve self-efficacy of behavior change and set SMART goals each month [31].

### Data Collection and Management

#### Data Collection Overview for Primary Participants

Demographics collected will include age, sex, race/ethnicity, marital status, and insurance status. In addition, 24-hour dietary recalls will be used to assess dietary intakes throughout the intervention by the research dietitian. The recalls will include 2 weekdays and 1 weekend day. Dietary recalls will be entered into the Automated Self-Administered Dietary Assessment (ASA-24) tool managed by the National Cancer Institute. ASA-24 uses a multipass method for capturing dietary recall data. From the collected data, Healthy Eating Index-2015 (HEI-2015) scores will be calculated. The HEI-2015 scores (0%-100%) will depict the quality of the diet in comparison to the Dietary Guidelines for Americans (DGA), with a higher percentage indicating higher alignment with DGA recommendations.

Validated surveys will be used to assess self-care activities and psychosocial factors pre- and postintervention. The International Physical Activity Questionnaire (IPAQ) short form will be used to collect physical activity data. [32] The IPAQ is a validated, self-reported, 6-item tool that collects weekly activity, resulting in a total activity score per week in Metabolic Equivalent Task (MET) minutes, with further categorization of high, moderate, or low activity levels. The following diabetes-related psychosocial factors and behaviors will also be collected through validated tools:

- Social support: The Medical Outcomes Study (MOS) Social Support Survey uses a 5-point Likert scale to score items related to perceived social support [33], and the Friends and Family Involvement in Adults’ Diabetes is a 16-item measure that assesses how helpful and harmful family members’/friends’ involvement in adults’ T2DM self-care is [34].
- Diabetes distress: The Diabetes Distress Scale (DDS) is a 17-item scale that measures patient concerns about disease management, support, emotional burden, and access to care. The response scale for each question ranges from 1 (“not a problem”) to 6 (“a very serious problem”). An average score of ≥3 indicates moderate distress and discriminates between high- and low-distress groups [35].
- Empowerment/self-efficacy: The Diabetes Empowerment Scale is a 23-item scale that measures diabetes-related psychosocial self-efficacy and uses 3 subscales: Managing the Psychosocial Aspects of Diabetes, Assessing Dissatisfaction and Readiness to Change, and Setting and Achieving Diabetes Goals [36].
- Diabetes knowledge: The Diabetes Knowledge Questionnaire (DKQ) is a 24-item survey that has a reliability coefficient of 0.78 and shows sensitivity to a diabetes knowledge intervention. In addition, it targets knowledge deficits that can be related to measurable outcomes, false statements, or common or serious misconceptions [37].
- Diabetes self-care activities: The Diabetes Self-Management Questionnaire (DSMQ) is a 16-item questionnaire to assess self-care activities associated with glycemic control and has an internal consistency of 0.84 [38].

Participants will not be removed from the study for nonadherence to the study protocol. In addition, if any participant chooses to leave the study early, data collected until that point will remain in the study database and will not be removed, but the participant will be discontinued from the study activities, such as medical nutrition therapy (MNT) sessions, and the participant will not be compensated for any sessions after removal from the study. Finally, if a participant indicates that their participation in the study is causing them distress, the PI will telephone the participant within 24 hours to see how they are managing and may suggest they contact their primary care provider. In the event the participant does not have a primary care provider, the PI will offer them a list of appropriate referral sources if they wish. In addition, to avoid future incidents, each case of possible distress will be reviewed with the PI and appropriate members of the investigative team, including a review of study materials and responses to distressed participants. This review will evaluate whether any aspect of the study requires modification.

#### Data Collection Overview for Family Members

Demographics data for family members will include age, sex, race/ethnicity, marital status, and insurance status. Diabetes status (diagnosed diabetes, prediabetes, no diabetes) will be confirmed via point-of-care HbA_1c_ tests at baseline, and overweight/obesity will also be evaluated during baseline data collection. Family members will have the option to participate in MNT sessions, as well as attend the 4-week Dining with Diabetes program with the primary participants. Consistent with data collection of the primary participants, medications and anthropometric and point-of-care data will be collected at baseline, 3 and 6 months during the intervention phase, and 3 and 6 months postintervention. Data will be collected by the research coordinator and research dietitian, as outlined before.

### Statistical Analysis

#### Community Assets Social Setwork Analysis

We will use an egocentric approach of social network analysis to understand the availability of resources within a 10-mile radius of each participant’s home. Frequencies, proportions, and means will be determined, where appropriate, for each network for the following components: network size, closeness, and density. For each participant, 2 social networks will be mapped, perceived (participant) and objective (research coordinator).

#### Intergenerational/Familial Diabetes and Obesity Social Network Analysis

Frequencies, proportions, and means will be determined, where appropriate, for each network for the following components: network size, age, number of female alters in a network, number of males in a network, closeness, and proportion of the participant’s network who are overweight/obese and have been diagnosed with prediabetes or diabetes.

#### Analysis of Clinical Outcomes

HbA_1c_ and weight will be assessed for the primary study participants using a longitudinal linear mixed model. Linear mixed models, an extension of the general linear model, are suitable when the independence of observations assumption is violated, which occurs when individuals are measured repeatedly over time. The primary outcomes will be HbA_1c_ values and weight measured at the 4 postbaseline follow-up points, each separated by 3 months, with a specific focus on the first postintervention follow-up point. In this hypothesis, “clinically meaningful” is defined as a ≥0.5% reduction in HbA_1c_ or a ≥6% reduction in weight. The main fixed effect of interest will be the categorical predictor of time, centered at the baseline testing occasion. To better understand how HbA_1c_ and weight change across the duration of the study, a significant overall effect of time will be followed up by comparing the adjusted means at adjacent time points (eg, baseline vs 3 months, 3 months vs 6 months, and so on). Additionally, the nature of change over time will be further characterized through the inclusion of polynomial trends (eg, quadratic functions). These tests will be adjusted for multiple comparisons using the Benjamini-Hochberg adjustment [39]. The model will incorporate random effects to account for repeated measures of individuals using an autoregressive covariance matrix to model the random effects. Since this is an observational, nonrandomized study, a set of baseline covariates (eg, age, weight, sex, race/ethnicity) will be used to control for demographic and other background trait characteristics.

Anthropometric indicators of family members presenting with overweight/obesity or a sustained healthy body composition will also be tested using a longitudinal linear mixed model. The primary outcome will be weight, measured at the follow-up testing occasions, with a specific focus on the first postintervention follow-up point. The main fixed effect of interest will be the categorical predictor of time. This analysis will include an additional set of random effects in addition to existing ones to account for repeated measures of participants, since family members are nested within the household, modeled using an unstructured covariance matrix. This analysis will include covariates at both the household (eg, family structure characteristics) and the individual (eg, age, gender) level to control for participant and household trait factors.

## Results

This study was funded in November 2022 by a Nutrition & Diabetes Innovative Clinical or Translational Science (ICTSN) Research Award through the ADA, who played no role in the study design; collection, management, analysis, or interpretation of data; writing of this report; or the decision to submit for publication. Data collection started on September 1, 2023, and is projected to end in November 2025. A total of 48 participants have been enrolled, and recruitment has ended; in addition, 14 (29%) of those participants have completed the intervention.

## Discussion

### Summary of Anticipated Findings

It is expected that participants will have a clinically meaningful change in HbA_1c_ (≥0.5%) and weight (≥–6%), as they will have participated in MNT and Dining with Diabetes, both of which have been shown to improve diabetes-related clinical outcomes [25,26,28]. It is also hypothesized that members of the participants’ households will have improved anthropometric indicators of overweight/obesity or maintain a healthy weight, as they are participating in the intervention and as the use of family-based interventions related to adult chronic diet-sensitive diseases have been associated with positive health-related outcomes [17-19].

### Strengths and Limitations

One strength of this study is the use of a family-based intervention to assist participants diagnosed with T2DM, as these interventions can facilitate health eating and physical activity in both participants and family members. Another strength of this study is that study activities are conducted in the home environment or at Extension offices, providing a more accurate representation of the family system. Lastly, combining MNT, a clinical approach, with Extension, a community-based approach, to offer chronic disease management education is a strength as it allows for an opportunity to translate research into practice and provide positive behavior change.

The limitations of this study include the pre-post design, which leads to difficulty in establishing causality, and other confounding variables could be contributing to the outcomes. Another limitation is that these results are from rural Kentucky participants and may not be generalizable to other rural areas within the United States or nationally.

### Conclusion

This is a feasibility study, so there is no need to randomize participants to the intervention. Data collected on the feasibility and acceptability of leveraging family units as sources of social support with MNT and nutrition education through the Dining with Diabetes program, along with the intervention workflow and efficacy, will help refine the research for a large-scale study that would be randomized to show the intervention’s effectiveness. The study-related findings will be presented at scientific meetings and published in peer-reviewed journals.

## Appendix

Multimedia Appendix 1 Peer review report by the American Diabetes Association.

## Abbreviations

ADA: American Diabetes Association
ASA-24: Automated Self-Administered Dietary Assessment
DDS: Diabetes Distress Scale
DGA: Dietary Guidelines for Americans
DKQ: Diabetes Knowledge Questionnaire
DSMQ: Diabetes Self-Management Questionnaire
Extension: Cooperative Extension Service
HbA_1c_: glycated hemoglobin A_1c_
HEI-2015: Healthy Eating Index-2015
IPAQ: International Physical Activity Questionnaire
IRB: Institutional Review Board
MNT: medical nutrition therapy
MOS: Medical Outcomes Study
PI: principal investigator
SMART: specific, measurable, achievable, relevant, and time-bound
T2DM: type 2 diabetes mellitus

## Data Availability

BLS will have access to the final trial dataset and study-related materials. All data resulting from the American Diabetes Association–funded research that can be shared without compromising human subject protections will be shared to an approved open data repository within 6 months of publication or within 18 months of the conclusion of the funding period, if the study remains unpublished. The datasets generated or analyzed during this study are available from the corresponding author upon reasonable request.
