# Supplementary material for: Promoting Intergenerational Health in Rural Kentuckians With Diabetes (PIHRK’D): Protocol for a Longitudinal Cohort Study
Source: JMIR Res Protoc. 2025 Jul 24;14:e69301. doi: 10.2196/69301 (PMC12332449; doi:10.2196/69301)
Supplement: Multimedia Appendix 1 [file resprot_v14i1e69301_app1.pdf]

American Diabetes Association®

2451 Crystal Drive, Suite 900
Arlington, VA 22202
1-800-DIABETES (800-342-2383)

ADA Peer Grant Review

Applicant Name: Brittany L. Smalls, PhD
Applicant Institution: University of Kentucky
Project Title: Addressing intergenerational obesity and promoting healthy eating and physical activity among individuals living with diabetes in Appalachia Kentucky
Requested Amount: $596,981.00
Program Area: Health Disparities
Diabetes Type: Type 2 Diabetes
Therapeutic Goal: Manage Diabetes

Review 1

Research Seeks to Address: As stated in the proposal, "the overarching hypothesis is that by utilizing a multifaceted approach to addressing intergenerational obesity and promoting healthy eating and physical among individuals living in rural Appalachia while also accounting for social environmental factors, lifestyle changes will be adopted and sustained over time."

Overall Evaluation: The majority of the weaknesses appeared to have been adequately addressed in the revised proposal, based on reviewer comments. As noted before, Extension offices in Kentucky are located in all 120 counties, which will help in the dissemination of appropriate educational materials and programs in all counties.

Research Improvements: It says, "Extension has over 1000 years of experience meeting the needs of rural communities in varying capacities." Should this be "100 years" instead?

Duplicative Research: The researchers claim that for their area of Appalachia (Kentucky), this type of research has not been done. Given their greater familiarity with the literature on this topic, their claim is likely valid.

Impact: The Appalachia area of Kentucky has higher rates of obesity and type 2 diabetes than most other areas of the country. If such a program can result in significant improvements in weight and A1C levels in participants, then it can be rolled out to the rest of the population there to help bring about better health.

Clear Conclusion: Since the only true outcomes are weight and A1C, it will be possible to conclude whether those have changed (assuming the study is adequately powered as indicated).

Timeframe: Yes, it appears doable as proposed.

Innovative and Incremental: Innovative, as this has not been done through the Extension offices before.

PI Qualifications: Yes, the PI and the rest of her team are well versed in the research area and appear capable of performing the work proposed.

Institutional Support: Yes, and the Extension offices in Kentucky give them additional resources to conduct the study.

Ethical Concerns: N/A

American Diabetes Association®

2451 Crystal Drive, Suite 900
Arlington, VA 22202
1-800-DIABETES (800-342-2383)

Review 2

Research Seeks to Address: The overarching hypothesis is that by utilizing a multifaceted approach to addressing intergenerational obesity and promoting healthy eating and physical among individuals living in rural Appalachia while also accounting for social environmental factors, lifestyle changes will be adopted and sustained over time. The hypotheses below correspond to AIM 3. 1. Participants (overweight/obese individuals living with diabetes) will have a clinically meaning change in hemoglobin A1c (≥ 0.5%) and weight (≥ - 6%) 2. Members of the households will have improved anthropometric indicators of being overweight/obese or maintain a healthy weight.

Overall Evaluation: Major strengths: 1. The project has great potential to impact poor outcomes for individuals with type 2 diabetes living in rural Kentucky. 2. Utilizes community based/placed resources that are already vetted by the community. This will increase the chance of sustainability of the program. 3. Intervening at the level of the family could lead to improvements in outcomes across generations. 4. Excellent team with credentials and experience necessary to carry out the study. 5. The Dine in program is accessible via video/telephone. Major weaknesses: 1. In home delivery of a major component of the intervention may limit future sustainability as this mode of delivery is expensive and requires a significant amount of resources. 2. Although the primary outcome for the study is change in A1c and weight for the primary participant living with diabetes a part of Aim 3b is focused specifically on changes for household members. The study team acknowledged that family members may not be engaged. However, there is no contingency plan to try to address low engagement amongst household members. One of the most innovative aspects of this study is the possibility that intervening in the household with an individual living with diabetes could have a positive effect on family members who are at risk. Acknowledging the limitations without planning to address it has the potential to lead to failure of Aim 3b and presents as a missed opportunity to advance a gap in our knowledge about multigenerational intervention.

Research Improvements: 1. Explore the possibility of transition of the in-home intervention to a virtual (telephone/video) mode in cases where access to the home is temporarily or permanently limited. 2. Address limitation #2 with simple strategies to enhance engagement of family members.

Duplicative Research: Other research groups are working in rural environments. Very few are proposing to utilize state-wide embedded networks to deliver interventions that can have an impact on diabetes outcomes.

Impact: The proposed work has the potential to impact a family unit which can have impact on multiple individuals at different stages of life.

Clear Conclusion: Yes. The experimental approach proposed will allow for clear conclusions.

Timeframe: Yes. I have no concerns about the timeline proposed.

Innovative and Incremental: The proposed research is innovative for two reasons: 1) The research will allow for exploration of the potential effect of the intervention on household members and 2) Leverages unique state-wide programs for intervention delivery in rural communities.

PI Qualifications: Yes. The applicant and team are well positioned to perform the work proposed.

Institutional Support: Yes. I have no concerns about the resources available to the investigator. Ethical Review

Ethical Concerns: N/A
